# Supplementary material for: Synthesis of ZnxCd1-xSe@ZnO Hollow Spheres in Different Sizes for Quantum Dots Sensitized Solar Cells Application
Source: Nanomaterials (Basel). 2019 Jan 22;9(2):132. doi: 10.3390/nano9020132 (PMC6410306; doi:10.3390/nano9020132)
Supplement: Supplementary file 1 [file nanomaterials-09-00132-s001.pdf]

Supplementary Materials

Synthesis of $Zn_xCd_{1-x}Se$@ZnO Hollow Spheres in Different Sizes for Quantum Dots Sensitized Solar Cells Application

Libo Yu [1] and Zhen Li [1,2,*]

[1] College of Chemistry and Chemical Engineering, Hexi University, Zhangye City 734000, Gansu Province, China; yulibo_665@163.com
[2] Key Laboratory of Hexi Corridor Resources Utilization of Gansu, Hexi University, Zhangye City 734000, Gansu Province, China; lizhen_665@163.com
* Correspondence: lizhen_665@163.com

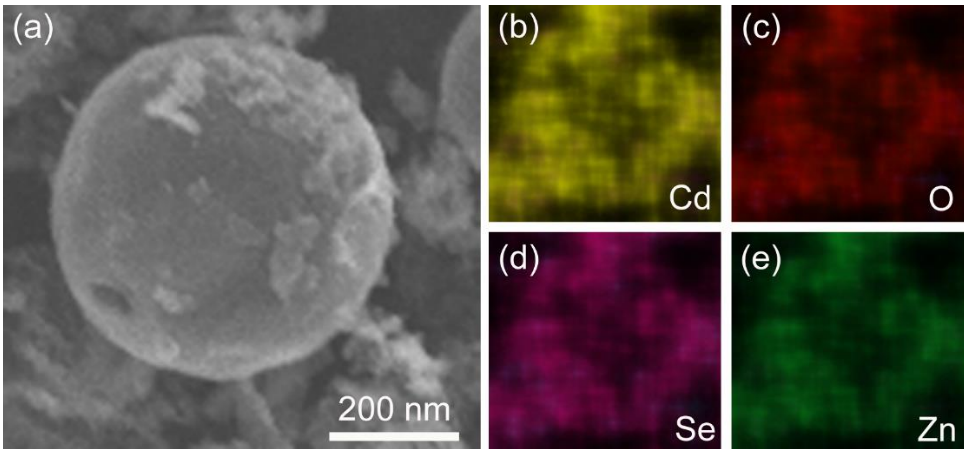

Figure S1. (**a**) SEM image $Zn_xCd_{1-x}Se$@ZnO hollow sphere; (**b–e**) elemental mapping of $Zn_xCd_{1-x}Se$@ZnO hollow sphere.

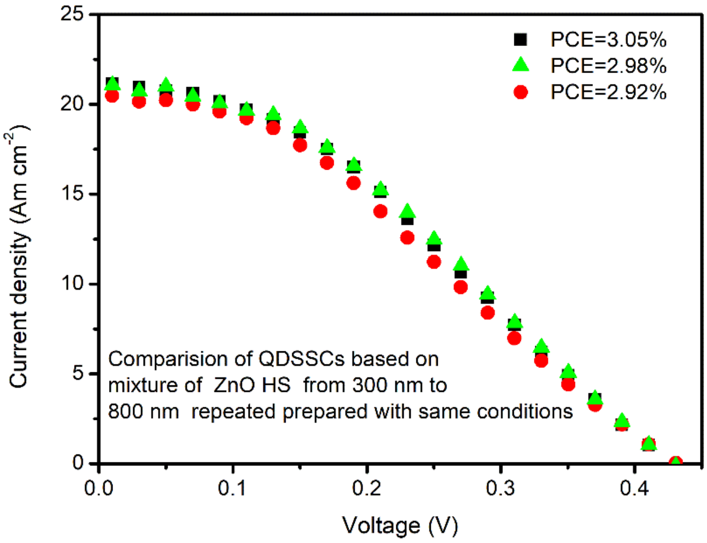

Figure S2. I–V curves QDSSCs based on mixture of ZnO HS from 300 nm to 800 nm (three QDSSCs are repeatedly prepared with same conditions for comparisons and reproducibility check).
